# Computed Tomography Findings of Children Under 3 Years of Age with Mild Traumatic Brain Injury (TBI) and No Neurological Focal Signs

**DOI:** 10.3390/jcm14082728

**Published:** 2025-04-16

**Authors:** Ksenija Markovic, Goran Djuricic, Djordje Milojkovic, Dusan Banovac, Kristina Davidovic, Dragan Vasin, Jelena Sisevic, Slavisa Zagorac, Boris Gluscevic, Dejan Bokonjic, Vuk Djulejic, Natasa Milic

**Affiliations:** 1 Institute for Medical Statistics and Informatics, Faculty of Medicine, University of Belgrade, 11000 Belgrade, Serbia; 2 Department of Diagnostic Imaging, University Children’s Hospital, 11000 Belgrade, Serbia; 3 Faculty of Medicine, University of Belgrade, 11000 Belgrade, Serbia; 4 Center for Radiology and Magnetic Resonance Imaging, University Clinical Center of Serbia, 11000 Belgrade, Serbia; 5 Clinic for Orthopedic Surgery and Traumatology, University Clinical Center of Serbia, 11000 Belgrade, Serbia; 6 Institute for Orthopedic Surgery “Banjica”, 11000 Belgrade, Serbia; 7 Department of Pediatrics, Faculty of Medicine Foca, University of East Sarajevo, 71123 Foca, Bosnia and Herzegovina; 8 Laboratory for Multimodal Neuroimaging, Institute of Anatomy, Faculty of Medicine, University of Belgrade, 11000 Belgrade, Serbia

**Keywords:** mild traumatic brain injury, CT infants, brain injuries, neuroimaging children

## Abstract

**Background/Objectives**: Mild traumatic brain injury (mTBI) is a leading cause of pediatric emergency department visits, particularly among children under three years old. Although computed tomography (CT) is the gold standard for diagnosing intracranial injuries, its use in young children poses radiation risks. Identifying reliable clinical indicators that justify CT imaging is essential for optimizing both patient safety and resource utilization. Objective: This study aimed to evaluate CT findings in children under three years of age with mTBI and no focal neurological deficits, as well as to identify clinical predictors associated with skull fractures and intracranial injuries. **Methods**: A retrospective analysis was conducted on 224 children under 36 months who presented with mTBI to a tertiary pediatric hospital from July 2019 to July 2024. Demographic data, injury mechanisms, clinical presentation and CT findings were evaluated. Univariate and multivariate regression analyses were performed to identify risk factors associated with skull fractures and intracranial injuries. **Results**: Falls accounted for 96.4% of injuries, with the majority occurring from heights of 0.5–1 m. The parietal region was the most frequently affected site (38%). Skull fractures were present in 46% of cases and were primarily linear (92.8%). Intracranial hematomas were identified in 13.8% of cases, while brain edema was observed in 7.6%. Significant predictors of skull fractures included age under 12 months (*p* < 0.001), falls from 0.5–1 m (*p* = 0.005), somnolence (*p* = 0.030), scalp swelling (*p* = 0.001) and indentation of the scalp (*p* = 0.016). Parietal bone involvement was the strongest predictor of both skull fractures (OR = 7.116, *p* < 0.001) and intracranial hematomas (OR = 4.993, *p* < 0.001). Conversely, frontal bone involvement was associated with a lower likelihood of fractures and hematomas. **Conclusions:** The findings highlight key clinical indicators that can guide decision-making for CT imaging in children with mTBI. Infants under 12 months, falls from moderate heights and parietal bone involvement significantly increase the risk of fractures and intracranial injuries. A more refined diagnostic approach could help reduce unnecessary CT scans while ensuring the timely identification of clinically significant injuries.

## 1. Introduction

Head trauma is considered one of the leading causes of morbidity and mortality in the pediatric population [1,2,3]. One out of ten children will experience a traumatic head injury before their second year of life, with infants under six months of age being at higher risk for intracranial injuries [4]. However, the majority of children who seek medical attention for head injuries in the emergency room have mild head injuries, and falls account for injury causes in up to 96% of children aged up to 2 years [2]. Mild traumatic head injuries resulting from falls are often present with no symptoms or with mild symptoms and signs. However, this type of injury ranks among the top causes of death in children up to 4 years of age [5].

A mild traumatic brain (mTBI) injury is defined as one in which the child presents without any focal neurologic signs, responds to voice or touch, and has a level of consciousness > 13 on the Glasgow Coma Scale (GCS) [5,6]. Traffic accidents involve a different mechanism from that of a mild TBI; falls from the child’s own height or from a maximum of a 1.5 m are the most common cause. Given that traumatic head injuries resulting from falls are the leading cause of hospitalization in the pediatric population, a detailed diagnostic process must be conducted to determine the extent of the injury [7]. However, mild traumatic head injuries in the pediatric population pose a significant challenge, particularly in children under 3 years of age. Infants, in particular, present diagnostic difficulties due to their underdeveloped verbal abilities at that age [6].

Computed tomography (CT) is considered the gold standard in diagnosing traumatic head injuries in the pediatric population with its sensitivity and specificity approaching 100% [2,5,8]. However, CT also comes with certain risks: exposing such young children to radiation increases the risk of developing malignancies in adulthood [2,5]. The soft tissues of pediatric patients are still undergoing developmental processes, making them more susceptible to the effects of radiation exposure from computed tomography (CT). As a result, children may be more vulnerable to the development of severe ailments, including but not limited to leukemia and brain tumors [9]. Therefore, it is crucial to accurately identify the instances and situations where the diagnostic algorithm justifies the use of CT for children at such a young age [10].

For example, the Pediatric Emergency Care Applied Research Network (PERCAN) guidelines for the emergency evaluation and management of pediatric head trauma advocate for the administration of a head CT scan during the initial examination for children below the age of 2 exhibiting a GCS of 14 or other signs of altered mental status or palpable skull fracture [11,12,13]. Children with scalp hematoma, a history of loss of conciseness (LOC) ≥ 5 s, having a severe mechanism of injury, or not acting normally per the parent should undergo observation versus CT on the basis of physician experience. However, as most injuries fall in this category in children under 2 years old, physicians need more subtle protocols for this group of patients [14,15]. Given the inherent difficulty of taking medical histories with these patients, subtle signs such as lack of visual contact, irritability, somnolence, vomiting and acute crying take on greater importance. All these signs might be indicators of an increased likelihood of cranial fracture and/or intracranial injury. Therefore, this study aims to bridge this gap [16,17] by evaluating CT findings in children under 3 years of age with mTBI and no neurological focal signs while identifying key clinical predictors associated with skull fractures and intracranial injuries. In addition, a scoring system was proposed to refine the diagnostic protocols in pediatric emergency settings.

## 2. Materials and Methods

### 2.1. Study Population

This was a retrospective study of children under 36 months of age who presented to a tertiary care children’s hospital (The University Children’s Hospital in Belgrade) over a 5-years period (from July 2019 to July 2024) and who were referred to CT diagnostics for suspected skull fracture or intracranial injury. Patients with comorbidities such as hydrocephalus, febrile convulsions, craniosynostosis and arachnoid cysts were excluded from the study. This study excluded patients who sustained head injuries in traffic accidents, as these injuries were caused by a stronger force than the one being investigated. Head trauma resulting from the intensity of force in a traffic accident leads to injuries beyond the scope of our study, such as life-threatening conditions due to brain injury, skull fractures with complications and fatal outcomes. Birth injuries were also excluded. Finally, patients were excluded from this study due to missing clinical data that were part of our analysis.

### 2.2. Data Collection and Ethical Approval

For pediatric patients who obtained a CT scan of the head and entered the study cohort, demographic, trauma histories, symptoms, injuries and radiographic data were obtained. Heteroanamnestic data were collected from the parents about the clinical presentation of the children upon admission, including symptoms such as vomiting, loss of consciousness, drowsiness, fever and nosebleeds. Additionally, the presence of head swelling and concavity were considered. Neurological status upon admission, general condition, hospitalization, status during hospitalization and the presence of signs of elevated intracranial pressure were also documented. Patients’ head CT studies were systematically reviewed after they were obtained from the Picture Archiving and Communication System (PACS). The collected data were de-identified and treated with confidentiality. Informed consent for the use of CT for diagnostic purposes and its data/image use was obtained from the parents/guardians of each pediatric patient included in this study. Ethical approval was obtained from the Institutional Review Board (no: 017-16/19).

### 2.3. Mechanisms of Injury

The mechanisms of injury were classified as follows: (1) resulting from falls from beds, chairs, strollers, baby carriers or falling from parent’s arms, which would correspond to falls ranging from a height of 0.5 m to 1 m; (2) falls from stairs or falls from their own height, which would correspond to falls from a height of 0.5 m; (3) cases where objects fell on the child’s head; and (4) falling from climbing structures, bunk beds, ladders, slides and falling from a bicycle basket, which would correspond to falls from a height of 1 m to 2.5 m.

### 2.4. CT Imaging (CT)

Examinations were performed using computed tomography (SOMATOM go.Up, Siemens Healthineers, Erlangen, Germany), utilizing axial sections at intervals of 0.8 mm and 0.5 mm, with subsequent reconstructions in MPR (Multiplanar Reconstruction) and VRT (Volume Rendering Technique) modes. All CTs were independently reviewed by a board-certified radiologist (GDJ) with >10 years of experience in neuroradiology and musculoskeletal imaging in the pediatric population, blinded to the initial reports, who documented fracture features and intracranial injuries.

### 2.5. Statistical Analysis

Numerical data are presented as means with standard deviations or as medians with ranges. Categorical variables are summarized with absolute numbers with percentages. Outcome- and age-related differences in the prevalence of specific morphological substrates were assessed using the Chi-square test. In all analyses, the significance level was set at 0.05. Statistical analysis was performed using IBM SPSS statistical software (SPSS for Windows, release 25.0, SPSS, Chicago, IL, USA).

## 3. Results

A total of 224 patients under the age of three years were included in this study, with the majority (53.6%) being younger than 12 months. Falls were the most common cause of injury, with 62.9% occurring from heights of 0.5 to 1 m. Falls from standing height accounted for 19.2% of cases, while falls from greater heights (1–2.5 m) comprised 14.3%. Only 3.6% of injuries resulted from objects falling onto the child’s head. The parietal region was the most frequently affected site (38%), followed by the occipital (28.3%) and frontal (19.3%) regions. A smaller proportion of injuries involved the temporal region (2.7%) or multiple sites (11.8%). In terms of fall surfaces, the majority (85.3%) occurred on soft surfaces, while 14.7% involved hard surfaces (Table 1).

Vomiting (40.6%) and scalp swelling (67%) were the most common post-traumatic symptoms, while loss of consciousness (2.7%), somnolence (16.5%), epistaxis (2.7%) and fever (4%) were less frequently observed (Table 2).

**Figure 1:**
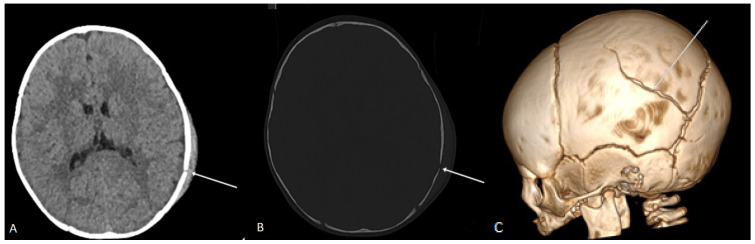
Axial plane CT scan in the soft tissue window (**A**) and bone window (**B**) of a 5-month-old child, showing a linear fracture of the left parietal bone with soft tissue edema and a subcutaneous hematoma of the scalp. In the VRT reconstruction, an arrow indicates the communication of the linear fracture of the left parietal bone with the lambdoid suture (**C**).

Skull fractures were present in 46% (103) of patients. Among these, 92.8% were linear fractures (Figure 1), 7.2% had fragmented fractures, and 9.8% presented with fracture dislocation. Brain edema was found in 7.6% of cases, intracranial hematoma in 13.8%, displacement of the midline structures in 0.9% and changes in the subarachnoid space in 11.2%. Additionally, changes in the ventricular system were noted in 8% of cases (Table 3).

### 3.1. Factors Associated with Skull Fractures, Intracranial Hematoma and Brain Edema

In our study population, children under 12 months had a significantly higher rate of skull fractures (73.8% of fractures occurred in this age group, *p* < 0.001), and falls from a height of 0.5–1 m were significantly associated with fractures (*p* = 0.005). Fractures were most frequently located in the parietal region (59.8%, *p* = 0.001). Frontal fractures were less common among fracture cases (6.9%, *p* = 0.001). Head swelling (*p* = 0.001), indentation of the scalp (*p* = 0.016) and vomiting (*p* = 0.001) were strongly associated with skull fractures. Somnolence was also significant (*p* = 0.030). Among children with head swelling, 75% had a fracture, whereas only 33.3% of children without swelling had a fracture. In total, 36.4% of children who vomited had a fracture, whereas 63.6% of children with fractures did not vomit (Table 4).

Our study identified the parietal region as the most common site associated with intracranial hematomas, accounting for 70% of cases (*p* = 0.001). Additionally, vomiting (*p* = 0.028), somnolence (*p* = 0.032) and scalp swelling (*p* = 0.010) were significant predictors of intracranial hematoma development. Children who fell from heights of 0.5–1 m had the highest likelihood of developing intracranial hematomas (71%), although this association did not reach statistical significance (Table 5).

Brain edema was diagnosed in 7.6% of cases, but no associations were found with the child’s age, gender or fall distance. Temporal injuries were significantly associated with brain edema (*p* = 0.011) (Table 6).

### 3.2. Univariate and Multivariate Regression Analysis for Fracture and Intracranial Hematoma as Dependent Variables

The outcomes from our study provide significant insights into the risk factors associated with skull fractures in patients who present with mTBI. Age under 12 months was identified as a strong predictor of skull fractures in young children with mild TBI. The univariate analysis showed that children under 12 months had a significantly higher odds ratio (OR = 4.926, 95% CI: 2.773–8.749, *p* < 0.001) of sustaining a skull fracture compared to those older than 12 months. The anatomical site of the injury played a crucial role in determining the fracture odds. Univariate analysis revealed that fractures were over 11 times more likely when the parietal bone was involved (OR = 11.159, 95% CI: 5.169–24.086, *p* < 0.001). Conversely, injuries involving the frontal bone were significantly less likely to result in a skull fracture (OR = 0.142, 95% CI: 0.058–0.346, *p* < 0.001). Compared to falls from standing height (used as a reference category), falls from 0.5–1 m and 1–2.5 m significantly increased the fracture odds in univariate analysis (OR = 3.306, 95% CI: 1.545–7.074, *p* = 0.002 and OR = 3.297, 95% CI: 1.243–8.440, *p* = 0.017). In multivariate analysis, a parietal bone injury was the most significant predictor of skull fracture, with the odds being only slightly reduced (OR = 7.116, 95% CI: 3.154–16.056, *p* < 0.001). Infants and fall from 1–2.5 m remained significant in multivariate analysis (OR = 3.096, 95% CI: 1.453–6.598, *p* = 0.003 and OR = 3.721, 95% CI: 1.186–11.672, *p* = 0.024) (Table 7).

Similar to the findings on skull fractures, the involvement of the parietal bone was the most significant predictor of intracranial hematoma. In both univariate and multivariate analyses, children with parietal bone injuries had a nearly fivefold increased odds of having an intracranial hematoma (OR = 4.993, 95% CI: 2.134–11.683, *p* < 0.001). In contrast, frontal bone injuries were associated with a significantly lower odds of having an intracranial hematoma (OR = 0.120, 95% CI: 0.016–0.914, *p* = 0.041). Scalp swelling was found to be a significant risk factor for intracranial hematoma, with a nearly fourfold increased odds in univariate analysis (OR = 3.841, 95% CI: 1.291–11.429, *p* = 0.016) (Table 8).

Based on the identified risk factors in our study, a scoring system was proposed to stratify children under 3 years of age with mild TBI and no neurological focal signs into risk categories for skull fractures. A point-based scoring system was derived from the ORs of significant predictors in the multivariate model (Table 9).

Each patient is assigned a cumulative score based on these risk factors, and they are classified into three risk categories. Low-risk patients (0 points) are unlikely to have a skull fracture, and clinical observation is recommended instead of immediate imaging. Moderate-risk patients (1 point) have a possible skull fracture, for which a skull X-ray should be considered as an initial screening with a selective CT scan if symptoms persist. High-risk patients (2 or more points) have a high probability of skull fracture, warranting an urgent CT scan for prompt diagnosis and management. This stratification system aims to optimize diagnostic and clinical decision-making by reducing unnecessary CT scans while ensuring the timely identification of significant injuries (Table 10).

## 4. Discussion

Traumatic brain injury is the most important cause of death and disability in children [18]. Its annual incidence is 150/100,000–450/100,000 worldwide [18]. However, the uncertainty surrounding the failure to accurately detect brain injury has resulted in the widespread and potential overuse of computed tomographic (CT) scanning in pediatric patients with minor head trauma, including those with a minimal possibility of intracranial injury [13]. Previous research indicates the potential feasibility of creating a decision tool that may effectively identify patients with head injury who possess a minimal risk of experiencing major cerebral injury. Consequently, using CT scanning in such cases may be regarded as unnecessary [19,20,21]. The utilization of the Pediatric Emergency Care Applied Research Network (PECARN) scale offers an efficacious approach to determine the necessity of a head CT scan for children; however, in this protocol, diagnostic management in terms of CT scan use for mild TBI is left to the physician’s experience [12,19]. As the majority of small children exhibit no clinically significant symptoms, i.e., present to the emergency room with a mild TBI, in this study, we aimed to accurately identify patients who truly require this diagnostic study to prevent unnecessary radiation exposure.

Head injuries are frequently encountered in pediatric practice but are typically not considered a major health concern from a treatment perspective [7,22,23]. Despite the occurrence of intracranial injuries and skull fractures, neurosurgical intervention is required in only a limited number of cases [2,5,24]. In our study, conservative treatment was the primary management approach. Although mild head trauma in children often resolves without lasting effects [5,7], the possibility of intracranial complications underscores the need for careful evaluation and management [24]. However, although the majority of these children do not necessitate additional testing, pediatric patients with minor head trauma might require a head computed tomography scan in order to exclude the possibility of a clinically significant traumatic brain injury.

In this study, we observed the following findings: among the 224 children under three years of age included in this study, injuries occurred more frequently at younger ages, with the highest incidence of fractures observed in infants aged <1 year; the most common fracture site was the parietal skull, followed by the occipital; and the leading cause of injury was fall from a height of 0.5–1 m (such as falling from a bed, table or chair). Our study also demonstrated that parietal bone fractures are the strongest independent predictor of intracranial hematomas, with a nearly fivefold increased risk. These findings emphasize the importance of assessing parietal bone involvement when evaluating pediatric head trauma [12]. Considering that parietal bone involvement is a common predictor of skull fractures and intracranial hematomas, incorporating skull X-rays into the initial diagnostic workup may aid in early detection and appropriate management. However, clinicians should be aware of the limited sensitivity of skull X-rays, as studies have reported sensitivities ranging from 63% to 71% for detecting single linear fractures. Therefore, while skull X-rays can be a useful initial tool, further imaging with CT scans may be warranted in cases where clinical suspicion remains high despite negative X-ray findings [25,26]. 

Male patients constituted the majority of the sample in our study (118 out of 224), a trend that is consistent with findings from previous research [22,23]. Among male participants, 51.7% presented with confirmed fractures, compared to 39.7% in the female group based on CT findings. The predominance of skull fractures among male patients aligns with the results of other studies exploring this association [5,27]. Clinical findings such as vomiting, somnolence, head swelling and scalp indentation have demonstrated statistically significant associations with fractures identified on CT scans. Among the 91 patients who experienced vomiting, 23 (25.3%) had confirmed fractures, while 68 (74.7%) did not. Conversely, Nee et al. [28] concluded that post-traumatic vomiting increases the risk of skull fracture by fourfold, although their study included both pediatric and adult populations, with no statistically significant differences between these groups. Da Dalt et al. argue that vomiting in children following head trauma is more likely associated with a personal or familial predisposition rather than serving as a clinical indicator for intracranial lesions [29]. Similar conclusions were drawn by Osmond et al., whose study of clinically significant indicators for CT imaging in pediatric head injuries found no association between vomiting and intracranial injuries. Instead, they identified factors such as a GCS score below 15 within two hours, suspected depressed skull fracture, worsening headache, persistent irritability, skull base fracture signs, subgaleal hematoma and severe injury mechanisms as significant predictors [30]. According to a study conducted by Lois K and colleagues, it was shown that children who experienced isolated loss of consciousness had a significantly reduced likelihood of suffering from significant traumatic brain injury. As a result, standard CT evaluation is not considered necessary for such cases [31]. Among the 150 patients presenting with head swelling, 89 (59%) were diagnosed with a fracture, suggesting that head swelling is a predictor of fracture presence. However, Beaudin et al. [5] reported contrasting results, indicating that none of the patients with skull fractures showed clinical symptoms such as nausea, vomiting, headache, loss of consciousness or convulsions. Furthermore, their study identified head hematoma as the most significant predictor of intracranial injury in children under the age of two. Hugenholtz et al. found that vomiting following minor head trauma is more prevalent among children older than two years. This observation is attributed to the incomplete myelination of brain neurons and ongoing brain maturation in this age group. In contrast, children under the age of two possess open cranial sutures, which allow for a greater dissipation of mechanical forces compared to closed sutures, thereby reducing the impact of trauma [31].

Among all patients with confirmed skull fractures on CT scans, 22 (9.8%) had dislocations. The majority of fractures were linear (92.8%), while seven fractures were multifragmentary. However, the important finding of our study is that skull fractures are significantly more common in children under 12 months of age. Claydon et al. [32] highlighted that even minor falls can result in severe head injuries, particularly concerning in infants due to the pliability of their skulls, which increases their vulnerability to skull fractures and intracranial injuries [33]. Mulligan et al. [34] noted that 32% of infants with epidural hemorrhages had fallen from a bed or sofa. Similarly, Warrington et al. [35] reported that falls occur in 22% of infants, although these incidents generally result in mild injuries primarily affecting the head, with serious outcomes such as concussions and fractures occurring in less than 1% of cases. Conversely, a study from China found that 71.16% of skull fractures in infants were associated with intracranial injuries, with a significant proportion of the 105 infants requiring surgical intervention. The authors emphasized that due to the open cranial sutures, intracranial injuries may occur even in the absence of a skull fracture. Consequently, they recommended conducting head CT scans for infants following falls, as such imaging plays a critical role in the early assessment of pediatric trauma patients [36].

The strategy for preventing skull injuries in children under one year of age should include the following: (1) enhancing parent education, (2) emphasizing the importance of emergency surgeons acquiring advanced knowledge in orthopedics and neurosurgery, and (3) establishing pediatric trauma centers and specialized pediatric trauma teams. Parents of infants under one year should be especially vigilant in preventing injuries, as younger children are at higher risk, particularly those of mothers under 24 years old [37]. Increased parental supervision is essential for preventing domestic injuries. Effective injury prevention includes keeping children visible and accessible [38,39], as well as using safety measures such as guard rails, carpets, and safety straps. Most infants with accidental fractures will exhibit behavioral signs such as crying and screaming; however, they may not show immediate symptoms of head injury, such as vomiting or convulsions. Therefore, parents should remain vigilant for delayed symptoms. Educating parents on trauma risks, initial management and prevention strategies is crucial for minimizing harm and ensuring infant safety.

Infants should initially be treated in specialized children’s hospitals or general hospitals with pediatric trauma centers to meet their developmental needs. Adult specialists should receive training in pediatric trauma care to ensure proper treatment and referrals. Establishing dedicated pediatric trauma teams within hospitals can improve care efficiency, with experts in emergency medicine, orthopedics, surgery and anesthesiology enhancing outcomes for severe cases [40]. University Children’s Hospital in Serbia serves as a model, successfully implementing an inter-hospital transfer system with neonatal teams specializing in critical pediatric trauma care.

### 4.1. Limitations

It is important to note that specific factors associated with injury mechanisms other than falls in children, such as birth-related injuries and abusive head trauma, were excluded from this study. This might impact the generalizability of our results. Furthermore, this research was conducted at a singular tertiary care facility, which may restrict the applicability of its results to other healthcare environments with varying patient demographics and clinical methodologies. The exclusion of patients with traffic accident-related injuries and other pre-existing neurological diseases, although justifiable, may result in an underrepresentation of more severe TBI cases, thus affecting the generalizability of the findings. While CT remains the conventional imaging technique for TBI evaluation, its associated dangers, including radiation exposure risks, are well documented. This study did not investigate alternate diagnostic methods, such as point-of-care ultrasound, which has shown potential in assessing juvenile head trauma. Future research should consider comparing the effectiveness of different imaging modalities in pediatric mild TBI cases to optimize the diagnostic accuracy while minimizing radiation exposure. Additionally, this study focused primarily on CT findings and did not evaluate the potential role of skull X-rays as an initial screening tool for detecting fractures, particularly in cases involving parietal bone injuries. Given the limited sensitivity of skull X-rays in detecting single linear fractures, future research should explore its utility in combination with clinical assessment to improve early detection and management strategies for mild TBI in young children.

### 4.2. Future Directions

Prospective multicenter studies are needed to assess the impact of delivering risk estimations for clinically significant mild traumatic brain injuries in the pediatric population under 3 years of age, together with corresponding therapeutic recommendations, on the outcomes observed in emergency department settings. The implementation of an electronic clinical decision-making support tool may help in reducing the utilization of computed tomography (CT) scans in the emergency department for children who are at a substantial risk of experiencing clinically significant traumatic brain injuries. This reduction in CT use should be achieved without compromising patient safety. The implementation of a multimodal approach, which incorporates a clinical decision support system, in the clinical pathway for mild head trauma should result in consistent enhancements in adherence and a cautious but significant decrease in the utilization of CT scans. This improvement should be used across various general emergency departments, involving patients who were generally considered to be at low risk.

## 5. Conclusions

This study highlights critical risk factors associated with skull fractures and intracranial hematomas in children under three years old who present with mTBI. Falls, particularly from a height of 0.5–1 m, were the most common cause of injury, with the parietal region being the most frequently affected site. Younger children, especially those under 12 months, exhibited a significantly higher risk of skull fractures. Skull fractures were primarily linear, with parietal bone involvement being the strongest independent predictor of both fractures and intracranial hematomas. Conversely, frontal bone injuries were associated with a significantly lower likelihood of fractures and intracranial hematomas. Key clinical predictors of skull fractures and intracranial hematomas included scalp swelling, somnolence and indentation of the scalp. Brain edema, although less common, was significantly associated with temporal injuries. These findings underscore the importance of clinical evaluation and imaging in young children with head trauma, particularly infants under 12 months. The main contributions of this study include establishing a refined risk stratification model for pediatric mild TBI, identifying clinical predictors that can assist in decision-making for CT imaging and proposing a potential framework for reducing unnecessary radiation exposure while ensuring patient safety. Future work should focus on external validation of the risk model across multiple healthcare settings. Additionally, integrating alternative imaging techniques, such as point-of-care ultrasound, could provide a more comprehensive diagnostic approach. Comparative studies incorporating large-scale datasets and advanced visualization techniques are needed to further enhance the clinical applicability.

## Figures and Tables

**Table 1:** Demographic characteristics, fall characteristics and injury sites in studied population.

Variable	*n* (%)
Age	
≤12 months	120 (53.6)
>12 months	104 (46.4)
Gender	
Male	118 (52.7)
Female	106 (47.3)
Comorbidities	21 (9.4)
Fall distance	
from one’s own height	43 (19.2)
from a height of 0.5–1 m	141 (62.9)
from a height of 1–2.5 m	32 (14.3)
fall of an object on child	8 (3.6)
Injury site	
Parietal	71 (38)
Frontal	36 (19.3)
Occipital	53 (28.3)
Temporal	5 (2.7)
Combined	22 (11.8)
Fall surface	
Soft	191 (85.3)
Hard	33 (14.7)

**Table 2:** Prevalence of clinical symptoms in studied population.

Symptom	*n* (%)
Vomiting	91 (40.6)
Somnolence	37 (16.5)
Loss of consciousness	6 (2.7)
Epistaxis	6 (2.7)
Temperature	9 (4)
General condition, worsening	14 (6.3)
Scalp swelling	150 (67)
Indentation of scalp	16 (7.1)

**Table 3:** Prevalence and types of injuries in studied population.

Injury	*n* (%)
Fracture	103 (46)
Linear	90 (92.8)
Fragments	7 (7.2)
Dislocation	22 (9.8)
Brain edema	17 (7.6)
Displacement of mediosagittal structures	2 (0.9)
Changes in the subarachnoid space	25 (11.2)
Changes in the chamber system	18 (8)
Intracranial hematoma	31 (13.8)
Hospitalization	199 (88.8)

**Table 4:** Demographic and fall characteristics, injury site and clinical symptoms in studied population according to fracture.

Variable	Fracture		*p*
	No (*n* = 121)	Yes (*n* = 103)	
Age			
≤12 months	44 (36.4)	76 (73.8)	<0.001
>12 months	77 (63.6)	27 (26.2)	
Gender			
Male	57 (47.1)	61 (59.2)	0.070
Female	64 (52.9)	42 (40.8)	
Fall distance			
from one’s own height	32 (26.4)	11 (10.7)	0.005 *
from a height of 0.5–1 m	66 (54.5)	75 (72.8)	
from a height of 1–2.5 m	15 (12.4)	17 (16.5)	
fall of an object on child	8 (6.6)	0 (0.0)	
Injury site			
Parietal	10 (11.8)	61 (59.8)	0.001
Frontal	29 (34.1)	7 (6.9)	0.001
Occipital	29 (64.1)	24 (23.5)	0.110
Temporal	4 (4.7)	1 (1)	0.116
>1 site	13 (15.3)	9 (8.8)	0.171
Fall surface			
Soft	103 (85.1)	88 (85.4)	0.947
Hard	18 (14.9)	15 (14.6)	
Vomiting	68 (56.2)	23 (22.3)	0.001
Somnolence	26 (21.5)	11 (10.7)	0.030
Loss of consciousness	3 (2.5)	3 (2.9)	0.841
Scalp swelling	61 (50.4)	89 (86.4)	0.001
Indentation of scalp	4 (3.3)	12 (11.7)	0.016

* *p* value derived after accounting for numerical limitations of statistical tests.

**Table 5:** Demographic and fall characteristics, injury site and clinical symptoms in studied population according to intracranial hematoma.

Variable	Intracranial Hematoma		*p*
	No (*n* = 193)	Yes (*n* = 31)	
Age			
≤12 months	100 (51.8)	20 (64.5)	0.188
>12 months	93 (48.2)	11 (35.5)	
Gender			
Male	101 (85.6)	17(14.4)	0.795
Female	92 (86.8)	14 (13.2)	
Fall distance			
from one’s own height	41 (21.2)	2 (6.5)	0.132 *
from a height of 0.5–1 m	119 (61.7)	22 (71.0)	
from a height of 1–2.5 m	26 (13.5)	6 (19.4)	
fall of an object on child	7 (3.6)	1 (3.2)	
Injury site			
Parietal	50 (31.8)	21 (70.0)	0.001
Frontal	35 (22.3)	1 (3.3)	0.016
Occipital	49 (31.2)	4 (13.3)	0.047
Temporal	4 (2.5)	1 (3.3)	0.807
>1 site	19 (12.1)	3 (10.0)	0.743
Fall surface			
Soft	167 (86.5)	24 (77.4)	0.184
Hard	26 (13.5)	7 (22.6)	
Vomiting	84 (43.5)	7 (22.6)	0.028
Somnolence	36 (18.7)	1 (3.2)	0.032
Loss of consciousness	6 (3.1)	0 (0)	0.320
Scalp swelling	132 (63.7)	27 (87.1)	0.010
Indentation of scalp	11 (5.7)	5 (13.8)	0.036

* *p* value derived after accounting for numerical limitations of statistical tests.

**Table 6:** Demographic and fall characteristics, injury site and clinical symptoms in studied population according to brain edema.

Variable	Brain Edema		*p*
	No (*n* = 207)	Yes (*n* = 17)	
Age			
≤12 months	112 (54.1)	8 (47.1)	0.575
>12 months	95 (45.9)	9 (52.9)	
Gender			
Male	109 (92.4)	9 (7.6)	0.982
Female	98 (92.5)	8 (7.5)	
Fall distance			
from one’s own height	43 (20.8)	0 (0.0)	0.306 *
from a height of 0.5–1 m	128 (61.8)	13 (76.5)	
from a height of 1–2.5 m	29 (14.0)	3 (17.6)	
fall of an object on child	7 (3.4)	1 (5.9)	
Injury site			
Parietal	65 (38)	6 (37.5)	0.968
Frontal	34 (19.9)	2 (12.5)	0.474
Occipital	49 (28.7)	4 (25)	0.756
Temporal	3 (1.8)	2 (12.5)	0.011
>1 site			
Fall surface			
Soft	179 (86.5)	12 (70.6)	0.076
Hard	28 (13.5)	5 (29.4)	
Vomiting	84 (40.6)	7 (41.2)	0.962
Somnolence	36 (17.4)	1 (5.9)	0.219
Loss of consciousness	6 (2.9)	0 (0)	0.477
Scalp swelling	138 (66.7)	12 (70.6)	0.741
Indentation of scalp	14 (6.8)	2 (11.8)	0.441

* *p* value derived after accounting for numerical limitations of statistical tests.

**Table 7:** Univariate and multivariate logistic regression analysis for skull fracture as dependent variable.

Variable	Univariate Regression			Multivariate Regression		
	*p*	OR	95% CI for OR	*p*	OR	95% CI for OR
Age ≤ 12 months	<0.001	4.926	2.773–8.749	0.003	3.096	1.453–6.598
Parietal bone	<0.001	11.159	5.169–24.086	<0.001	7.116	3.154–16.056
Frontal bone	<0.001	0.142	0.058–0.346			
Fall distance, own height	Ref.					
height of 0.5–1 m	0.002	3.306	1.545–7.074	0.135	2.032	0.801–5.156
height of 1–2.5 m	0.017	3.297	1.243–8.44	0.024	3.721	1.186–11.672

**Table 8:** Univariate and multivariate logistic regression analyses for intracranial hematoma as dependent variable.

Variable	Univariate Regression			Multivariate Regression		
	*p*	OR	95% CI for OR	*p*	OR	95% CI for OR
Parietal bone	<0.001	4.993	2.134–11.683	<0.001	4.993	2.134–11.683
Frontal bone	0.041	0.120	0.016–0.914			
Occipital bone	0.055	0.339	0.112–1.024			
Scalp swelling	0.016	3.841	1.291–11.429			
Indentation of the scalp	0.045	3.182	1.024–9.891			

**Table 9:** Risk score for predicting skull fractures in children under 3 years of age with mild TBI and no neurological focal signs.

Risk Factor	Assigned Score
Age ≤ 12 months	+1
Fall from 1–2.5 m	+1
Parietal bone injury	+2

**Table 10:** Scoring system for risk stratification for skull fractures in children under 3 years of age with mild TBI and no neurological focal signs.

Risk Category	Probability of Skull Fracture	Recommended Clinical Approach
Low Risk (0 points)	Unlikely to have a skull fracture or hematoma	Consider observation instead of imaging
Moderate Risk (1 point)	Possible skull fracture	Consider skull X-ray as initial screening, followed by selective CT if symptoms persist
High Risk (2+ points)	High probability of fracture	Urgent CT scan recommended

## Data Availability

The data presented in this study are available on request from the corresponding author due to privacy reasons.
